# Embryonic, Larval, and Juvenile Development of the Sea Biscuit *Clypeaster subdepressus* (Echinodermata: Clypeasteroida)

**DOI:** 10.1371/journal.pone.0009654

**Published:** 2010-03-22

**Authors:** Bruno C. Vellutini, Alvaro E. Migotto

**Affiliations:** 1 Centro de Biologia Marinha, Universidade de São Paulo, São Sebastião, São Paulo, Brazil; 2 Departamento de Zoologia, Instituto de Biociências, Universidade de São Paulo, São Paulo, São Paulo, Brazil; National University of Singapore, Singapore

## Abstract

Sea biscuits and sand dollars diverged from other irregular echinoids approximately 55 million years ago and rapidly dispersed to oceans worldwide. A series of morphological changes were associated with the occupation of sand beds such as flattening of the body, shortening of primary spines, multiplication of podia, and retention of the lantern of Aristotle into adulthood. To investigate the developmental basis of such morphological changes we documented the ontogeny of *Clypeaster subdepressus*. We obtained gametes from adult specimens by KCl injection and raised the embryos at 26

C. Ciliated blastulae hatched 7.5 h after sperm entry. During gastrulation the archenteron elongated continuously while ectodermal red-pigmented cells migrated synchronously to the apical plate. Pluteus larvae began to feed in 3 d and were 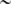
20 d old at metamorphosis; starved larvae died 17 d after fertilization. Postlarval juveniles had neither mouth nor anus nor plates on the aboral side, except for the remnants of larval spicules, but their bilateral symmetry became evident after the resorption of larval tissues. Ossicles of the lantern were present and organized in 5 groups. Each group had 1 tooth, 2 demipyramids, and 2 epiphyses with a rotula in between. Early appendages consisted of 15 spines, 15 podia (2 types), and 5 sphaeridia. Podial types were distributed in accordance to Lovén's rule and the first podium of each ambulacrum was not encircled by the skeleton. Seven days after metamorphosis juveniles began to feed by rasping sand grains with the lantern. Juveniles survived in laboratory cultures for 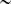
9 months and died with 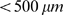 wide, a single open sphaeridium per ambulacrum, aboral anus, and no differentiated food grooves or petaloids. Tracking the morphogenesis of early juveniles is a necessary step to elucidate the developmental mechanisms of echinoid growth and important groundwork to clarify homologies between irregular urchins.

## Introduction

Sea biscuits and sand dollars are representatives of the order Clypeasteroida, the latest major branch of irregular echinoids to evolve. Clypeasteroids diverged from a cassiduloid-like ancestor around the Paleocene [1]–[3] and by Middle Eocene (49-37 million years ago) the suborders of this group were already established [2] and present in oceans worldwide [1]. Morphological changes during the early evolution of clypeasteroids were tightly associated with the occupation of shifting sand beds and involved the retention of juvenile traits into adulthood [2]. Strong evidence of this phenomenon is, for example, the presence of a lantern of Aristotle in adult clypeasteroids, while in the remaining extant and fossil irregular echinoids, including in the ancestral lineage of Clypeasteroida (a branch of Cassiduloida – see [3] for an analysis of cassiduloid-clypeasteroid relationships), the lantern atrophies before adult life [2]. Besides being the only irregular echinoids with the lantern of Aristotle and perignathic girdles in adult individuals, clypeasteroids exhibit more than one podium per ambulacral plate. This feature accounts for the drastic increase on the number of nonrespiratory tube feet and allows greater efficiency in the collection of food [2]. In addition, a recent study established the relatively large anus, shortening of primary spines, and flattening of the body as synapomorphies of Clypeasteroida [4].

The sea biscuit *Clypeaster subdepressus* (Gray, 1825) belongs to the suborder Clypeasterina, sister group of Scutellina, and to the Clypeasteridae, a single-genus family of Clypeasteroida and sister group of the Arachnoididae. *C. subdepressus* is found in the Caribbean and East Coast of America, from North Carolina to Rio de Janeiro [5] and São Paulo [6], [7]. Adult individuals live semi-buried in coarse biogenic sand of coastal waters and feed on organic matter present in the sediment [5], [8]. Sand particles are collected by accessory podia of the oral surface, taken to the mouth region, and then crushed by the lantern teeth [9].

The larval development and early juvenile growth of *C. subdepressus* was first described in Caribbean specimens [10]. The study compared the development of *C. subdepressus* and *C. rosaceus* showing that the former has an obligatory planktotrophic larva in contrast to the facultative feeding larva of the latter. Since this study focused on the characterization of *C. rosaceus* development, the timing of early development and a detailed ontogeny of morphological traits of *C. subdepressus* were not provided.

Given the unique morphological evolution, a complete fossil record [11], and readily feasible developmental studies [12], clypeasteroids can be a valuable group for evolutionary analyses. Furthermore, *C. subdepressus* has been used in recent studies related to development [13]–[16], posing the need for a detailed developmental table in order to comprehend the evolution of echinoid morphology. The present work provides a complete description of *C. subdepressus* development including stage timing, cleavage and gastrulation dynamics, and post-metamorphic development of juveniles.

## Results

The morphological changes occurring during the embryonic, larval, and juvenile development of *C. subdepressus* are depicted in Table 1.

**Table 1:** *C. subdepressus* developmental table.

Embryos			Larvae			Juveniles		
Events	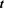		Events	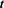		Events	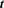	
Sperm entry	0		PMC	10	h	Lantern rudiments	0	d
Vitelline envelope elevation	30–40	s	Red-pigmented cells	12	h	Resorption of larval tissues	1	d
Fertilization membrane	2–6	min	SMC	13	h	Aboral plates	2	d
Pro-nuclei migration	5	min	Archenteron invagination	14–24	h	Gut differentiation	2–7	d
Pro-nuclei fusion	18	min	Larval skeleton	15	h	Anus	2	d
2 cells (96%)	80	min	Prism	24	h	Teeth ornamentation	4	d
4 cells (88%)	100	min	Coeloms	24	h	Aboral miliary spines	4	d
8 cells (74%)	120	min	Tri-parted gut	24	h	Pyramids	7	d
16 cells (88%)	160	min	Pluteus 2-arms	2	d	Mouth opening	7	d
32 cells (92%)	180	min	Feeding	3	d	Feeding	7	d
56–60 cells	3.2	h	Pluteus 4-arms	4	d	Ophicephalous pedicellariae	14	d
108 cells	3.5	h	Hidropore opening	5	d	Tridentate pedicellariae	30	d
Blastula formation	3.5–8	h	Vestibule	6–10	d			
Vegetal pole thickening	7.5–10	h	Pluteus 6-arms	6	d			
Cilia formation	7.5	h	Pluteus 8-arms	10	d			
Hatching	8	h	Rudiment	10–23	d			
			Metamorphosis	23	d			

Timing of morphological changes during embryonic, larval, and juvenile development of *C. subdepressus* at 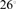
C. 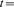
time after fertilization (Embryos and Larvae); 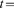
time after metamorphosis (Juveniles).

### Spawning

**Figure 1:**
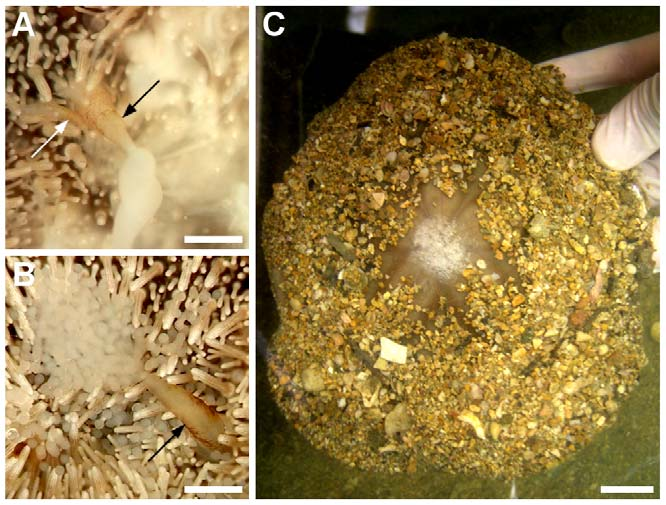
*C. subdepressus* genital papillae and spawning. **A** Male genital papilla (arrow) releasing sperm and adjacent accessory papilla (white arrow). **B** Female genital papilla (arrow) releasing eggs. **C** Adult male releasing sperm during a spontaneous spawning event in March 8, 2007. Scale bars = 1 mm (**A, B**); 20 mm (**C**)

Adult males and females of *C. subdepressus* had 5 gonopores at the apex. A long (2–3 mm) genital papilla (Fig. 1A,B) and up to 3 shorter (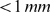) accessory papillae were present at the distal end of each gonoduct. During spawning induced by KCl injection one or two gonopores released gametes continuously for several minutes (maximum of 30 min with specimen underwater); the flux of gametes through a genital papilla varied between 7 and 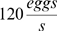 (Video S1). Accessory papillae of males released small quantities of sperm under low intensity flux (Fig. 1A; Video S2). A spontaneous spawning event was observed in the laboratory tanks on March 8, 2007 at 17 h 30 min. Five individuals moved the sand away from the apex and initiated spawning through the five gonopores simultaneously (Fig. 1C). Spawning ceased after 10 min and the apex was again covered with sand grains by action of surrounding spines. After a few minutes it was uncovered and gamete release re-initiated; this sequence was repeated at least 3 times for each specimen.

### Fertilization

**Figure 2:**
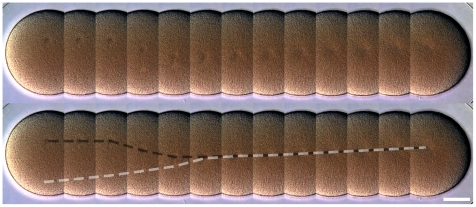
Migration and fusion of *C. subdepressus* pronuclei. Montage of a single zygote during a period of 12 min 30 s after sperm entry (top). The movements of male (black dashed line) and female (white dashed line) pronuclei were outlined (bottom). Frames taken every 58 s. Scale bar = 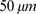

After sperm entry, the male pro-nucleus was pushed by microtubules towards the center of the egg (Fig. 2; Video S3). It moved at a constant speed of 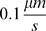. When touched by microtubules, the female pro-nucleus was rapidly pulled towards the male pro-nucleus. The fusion took place approximately 12 min 30 s after sperm entry. The cytoplasmic movements increased and the cell surface acquired an irregular aspect. Video footage accelerated 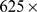 revealed that the deformations were dynamic and that the membrane was vibrating (Video S3). Immediately before the first cleavage the membrane ceased the vibration, the cell surface became regular, and the hialine layer thickened.

### Cleavages

**Figure 3:**
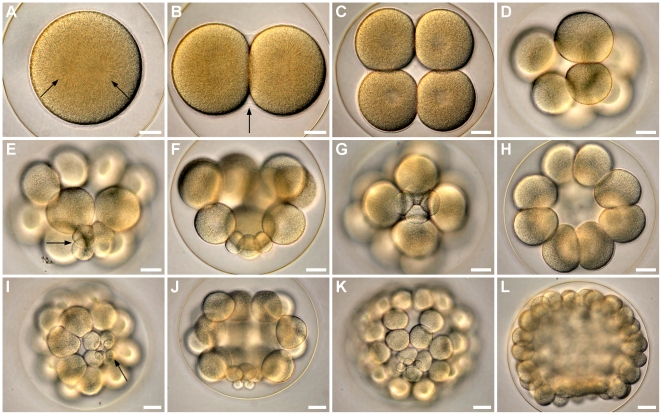
Early cleavages of *C. subdepressus* under light microscopy. **A** Two nuclei (arrows) during cariocinesis of the first cell division. **B** Embryo with two cells before the second cleavage; hialine layer is visible between cells (arrow). **C** Animal pole view of an embryo with 4 cells. **D** Lateral view of an embryo with 8 cells; blastomeres on the vegetal pole are smaller (bottom). **E** Micromeres (arrow) on the vegetal pole of an embryo with 16 cells. **F** Lateral view of an embryo with 16 cells. **G** Arrangement of micromeres and macromeres on the vegetal pole of a 16 cell embryo. **H** 16 cell embryo showing the mesomere arrangement on the animal pole. **I** Fifth division cycle showing child-micromeres (arrow) on the vegetal pole; embryo with 32 cells. **J** Lateral view of an embryo with 32 cells. **K** Vegetal pole of an embryo with 56 cells. **L** Lateral view of an embryo with 108 cells. Scale bars = 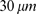

Cell divisions initiated 80 min after fertilization (Table 2) and were holoblastic. First and second cleavages were meridional, dividing the embryo in 4 equal blastomeres (Fig. 3A–C). The third division was equatorial, separating animal and vegetal blastomeres at 120 min; the latter were 30% smaller in diameter (Fig. 3D). Micromeres originated unequally from vegetal blastomeres while 8 mesomeres were formed by a meridional cleavage of animal blastomeres (Fig. 3E–H). Equatorial division of mesomeres, meridional division of macromeres, and unequal micromere division formed embryos with 32 cells 180 min after fertilization (Fig. 3I,J). Sixty-cell embryos were formed when blastomeres went through an equatorial division while the 4 micromeres experienced a meridional division. The seventh cleavage occurred without micromere division resulting in embryos with 108 cells (Fig. 3L).

**Table 2:** Dynamics of *C. subdepressus* cell divisions.

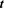	0	20	40	60	80	100	120	140	160	180
Eggs	**100.0**	4.1	4.9	5.1	1.4	1.7	2.0	0.4	2.1	2.2
Zygote	-	**95.9**	**95.1**	**92.9**	0.8	1.0	0.7	0.4	0.3	0.6
2 cells	-	-	-	1.9	**95.7**	7.9	1.0	-	-	-
4 cells	-	-	-	-	2.2	**88.0**	21.9	1.5	-	-
8 cells	-	-	-	-	-	1.4	**74.4**	45.2	9.7	0.3
16 cells	-	-	-	-	-	-	-	**52.5**	**87.9**	5.0
32 cells	-	-	-	-	-	-	-	-	-	**91.8**
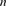	304	1145	411	311	369	291	301	259	331	317

Relative amount (%) of cell stages for each timespan during early cleavages. 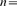
total embryos counted; 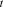 in min.

### Blastulae

**Figure 4:**
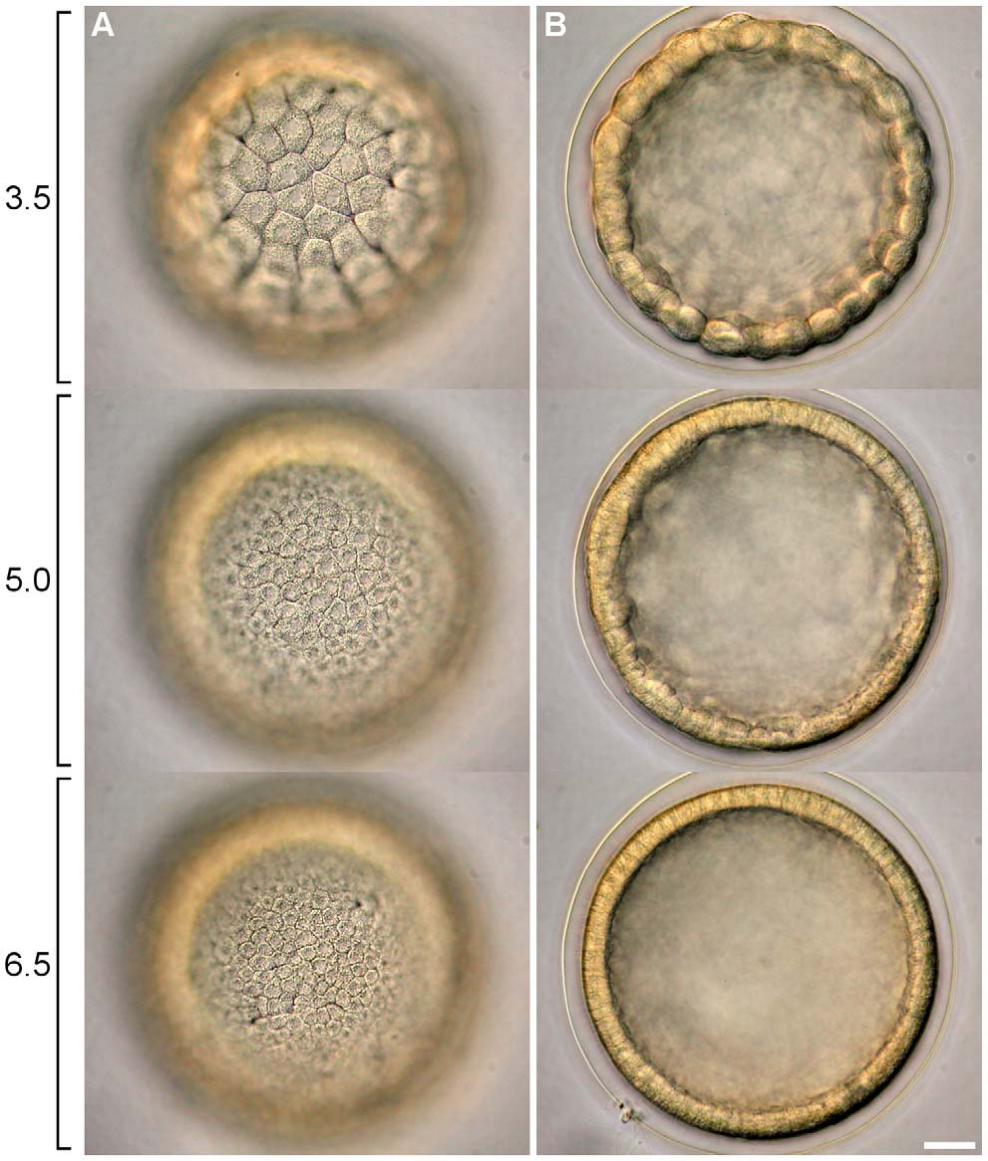
Compactation of ectodermic cells during blastula formation. **A** and **B** show different optical sections of embryos sampled 3.5, 5.0, and 6.5 hpf. **A** Ectodermic cells acquired a polygonal shape and became smaller during the division cycles. **B** Epithelium became more uniform and cells lost the globoid shape. Scale bar = 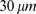

Cells acquired a polygonal shape during the consolidation of the epithelium between 3.5 and 6.5 h post fertilization (hpf) (Fig. 4). The vegetal plate thickened and cilia were formed 7.5 hpf, immediately before hatching.

### Gastrulae

**Figure 5:**
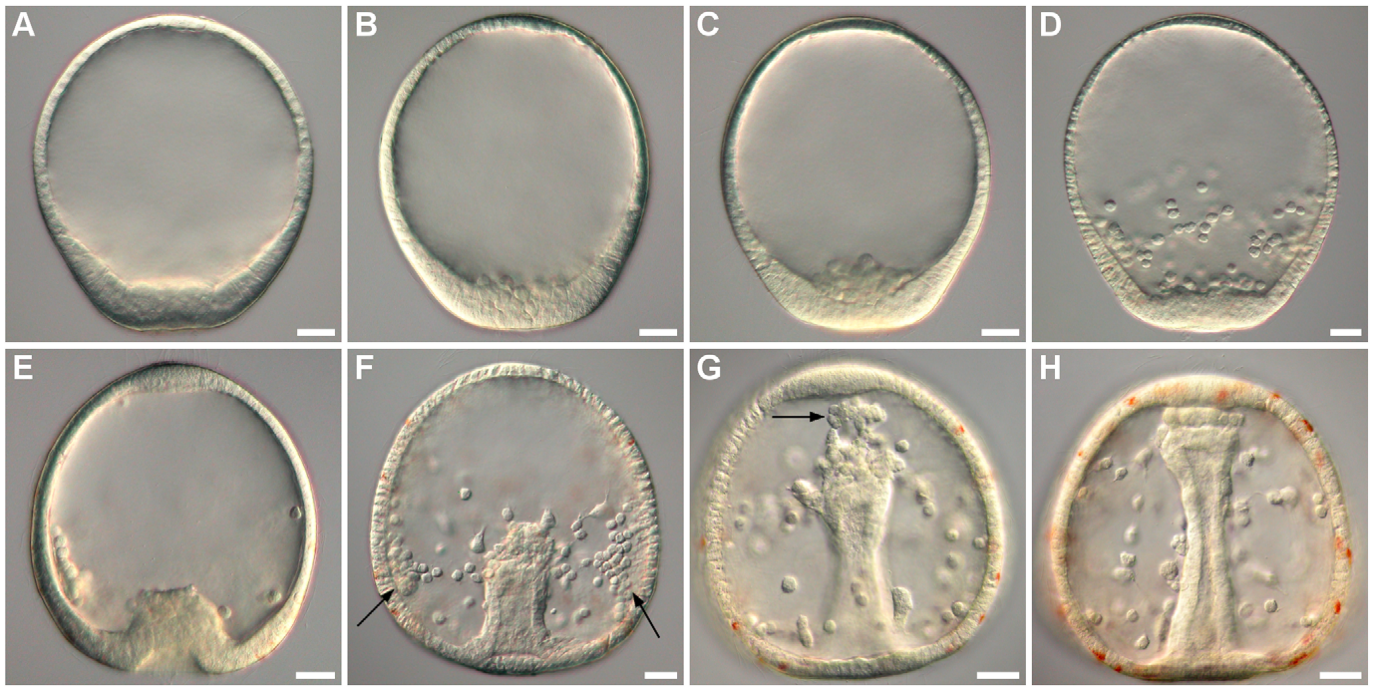
Sequential images of *C. subdepressus* embryos during gastrulation. Vegetal pole is bottom. **A** Post-hatching swimming blastula with thickened vegetal pole epithelium 9 hpf. **B** Initial ingression of PMC into the blastocoel 10 hpf. **C** Ingressed PMC aggregated on the posterior end 11 hpf. **D** Initial migration of PMC through the inner side of ectoderm 13 hpf; red-pigmented cells are present on the vegetal pole (not visible). **E** Initial invagination of the archenteron 14 hpf. **F** SMC ingress the blastocoel from the archenteron tip 15 hpf; PMC form lateral aggregates (arrows) and begin to secrete the skeleton. **G** SMC reaching the anterior pole 16.5 hpf (arrow); red-pigmented cells reached the middle region of the embryo through the ectoderm. **H** Final stage of archenteron invagination with SMC touching the anterior pole 19 hpf; blastocoel is populated with PMC and SMC and red-pigmented cells reached the anterior pole. Scale bars = 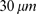

**Figure 6:**
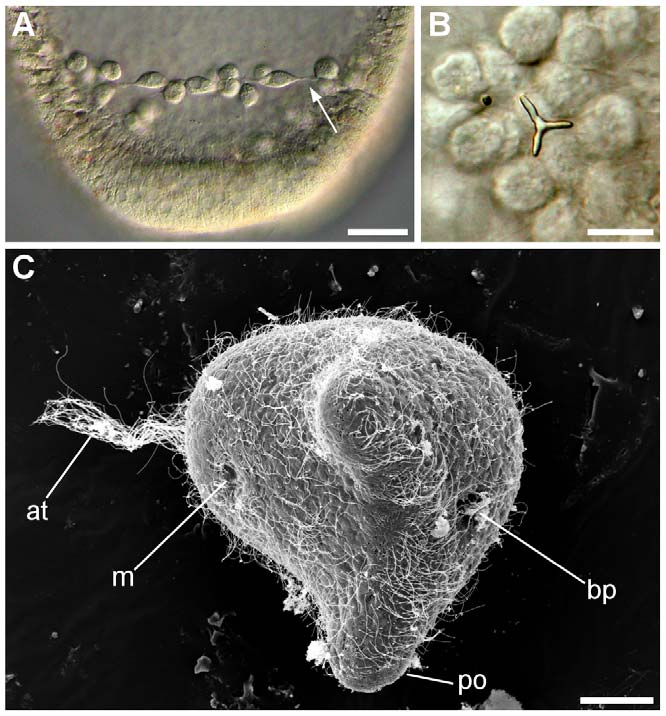
PMC and SEM of prism larval stage of *C. subdepressus*. **A** PMC forming a ring near the posterior end 14 hpf; cells are connected by cytoplasmic bridges (arrow). **B** Triradiate calcareous spicule secreted by PMC initiating the formation of the larval skeleton 15 hpf. **C** The apical tuft (at) and future mouth (m) are present at the anterior region. On the posterior end the postoral arms (po) begin to extend and the blastopore is visible (bp). Scale bars = 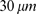 (**A, C**); 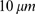 (**B**)

Primary mesenchyme cells (PMC) detached from the vegetal pole, became spherical, and aggregated in an unipolar manner on the vegetal pole; ingression to the blastocoel took place approximately 10 hpf (Fig. 5A–C). PMC migrated through the blastocoel forming a ring connected by thin filopodial pseudopodia on the posterior end (Fig. 6A).

Red-pigmented cells differentiated on the vegetal pole 12 hpf and migrated through the epithelium, simultaneously with PMC, towards the apical plate 13 hpf (Fig. 5D). Secondary mesenchyme cells (SMC) originated on the vegetal pole, extending cytoplasm projections towards the blastocoel during archenteron invagination. The archenteron invagination began at 14 hpf (Fig. 5E).

PMC formed two aggregates 15 hpf (Fig. 5F) and initiated the secretion of a calcareous triradiate spicule (Fig. 6B). SMC were composed of different cell types including cells with filopodial and lobopodial pseudopodia, ameboid cells without cytoplasm projections, and cells with red-pigmented granulae. SMC on the archenteron reached the anterior pole 16,5 hpf (Fig. 5G) while red-pigmented epithelial cells reached the anterior pole 19 hpf, when the blastocoel was occupied by SMC (Fig. 5H). Epithelial red-pigmented cells were not present on the ventral (oral) region of the embryo at prism stage. The surface of the embryo at prism stage was covered by cilia with an apical tuft on the anterior pole and a ciliated ring around the anus (Fig. 6C).

**Figure 7:**
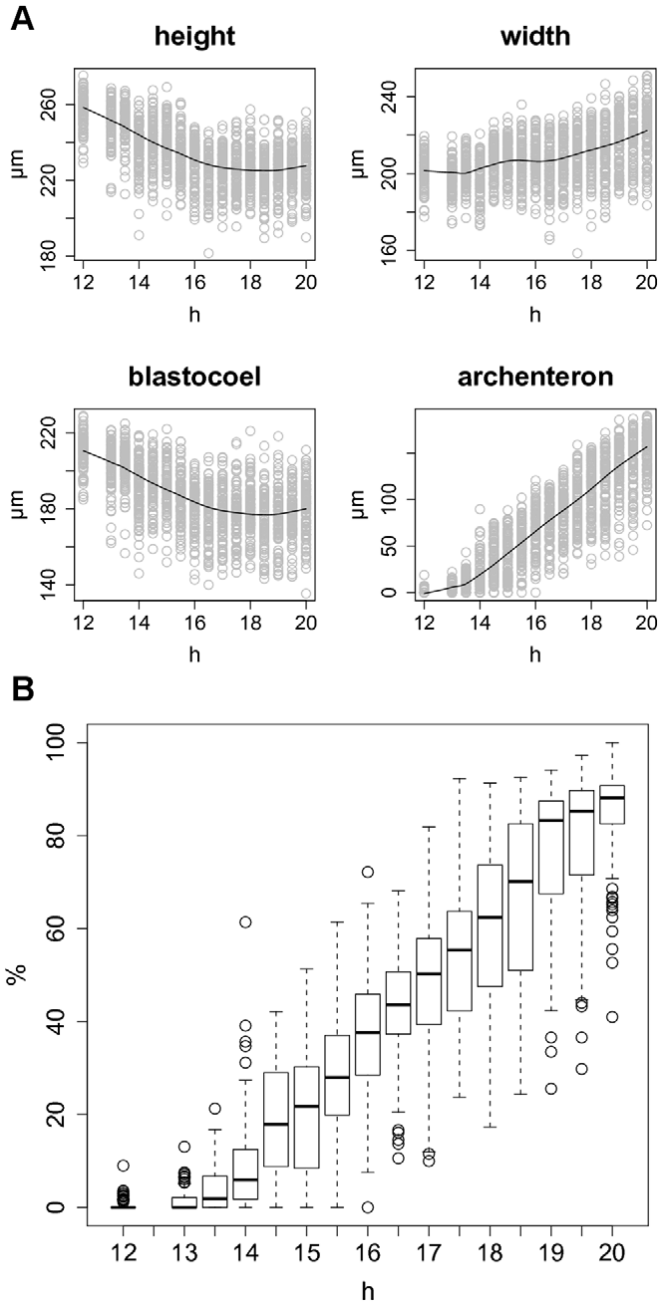
Gastrulation of *C. subdepressus*. **A** Time-series plots with fitted smooth curve of 4 morphometric measurements during gastrulation. The height, width, blastocoel height, and archenteron length were measured every 30 min for 8 h (12–20 hpf, except 12.5 hpf). Height and blastocoel height decreased during the period while the width showed a slight increase; archenteron elongation is continuous. **B** Relative amount of archenteron elongation during gastrulation calculated by the ratio between archenteron length and blastocoel height. 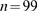 for each timespan.

The embryo height decreased during gastrulation from 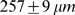 at 12 hpf to approximately 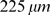 from 16.5 hpf to 20 hpf; the width had a slight increase of 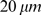 (Fig. 7). The archenteron elongated continuously on a linear manner from 13.5 hpf (Fig. 7) and reached 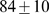
% of the blastocoel height (Fig. 7B).

### Pluteus

**Figure 8:**
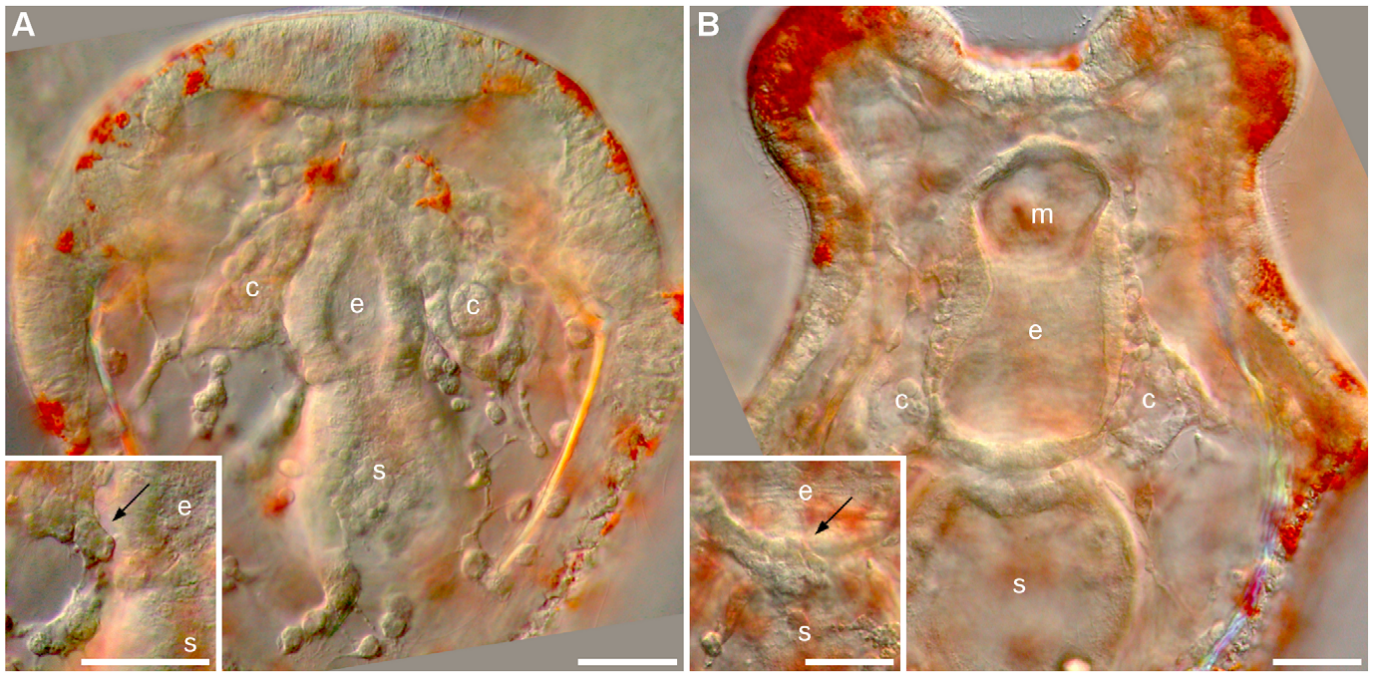
Coeloms, gut, and hydropore formation in *C. subdepressus*. **A** Differentiation of a three-part gut has begun and coelomic sacs (c) were formed next to the archenteron 48 hpf. Detail shows the initial extension of the hydropore channel (arrow) from the left coelom. **B** Differentiated gut, mouth (m), esophagus (e), and stomach (s), after 3 d. Hidropore channel (arrow) next to the larval epithelium 3 dpf. Scale bars = 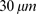

In 48 hpf two coelomic pouches were present next to the esophagus (Fig. 8A). At this stage the mouth opened, but the larvae were unable to feed; microalgae captured by the larval arms were carried towards the mouth, but were deflected away possibly by an opposing current (Video S4). The gut was not functional, but already had three portions identified as esophagus, stomach, and intestine (Fig. 8A). Muscles of the esophagus began to contract 70 hpf (Video S5); the stomach grew in diameter while its epithelium became thinner. The gut became functional and the larvae began to feed 3 d post-fertilization (dpf) (Fig. 8B).

**Figure 9:**
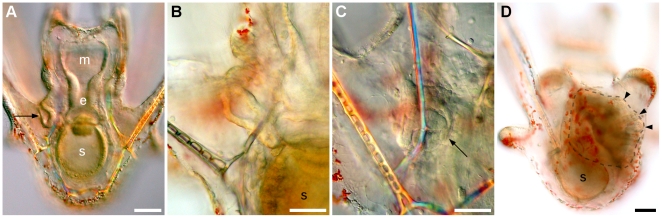
Vestibule and rudiment formation in *C. subdepressus* larvae. **A** Vestibule invagination 5 dpf (arrow). **B** Detail of vestibule reaching the left coelomic sac. **C** Fusion of vestibule and left coelom (arrow). **D** Posterior region of a competent pluteus larva showing a well-developed rudiment (dashed line). Appendages (podia and spines – arrowheads) are present and active; developing rudiment occupied most of the body and displaced the larval gut (s). Scale bars = 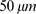 (**A, D**); 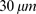 (**B, C**)

The skeleton of postoral and posterodorsal arms was fenestrated while the anterolateral and preoral pairs were non-fenestrated (see Fig. S1 for the arrangement of larval arms). The hydropore formed above the cardiac sphincter approximately 3 dpf by an extension of the left coelomic pouch and opened on the surface 5 dpf (Fig. 8A,B detail). The vestibule appeared between 4 and 5 dpf on the left side of larvae between the postoral and postero-dorsal arms (Fig. 9A). The vestibule fused with the left coelom (Fig. 9B,C) forming the rudiment (Fig. 9D) between 6 and 10 dpf.

The rudiment developed podia and spines, which became active still inside the larval body (Fig. 9D). No pedicellariae were formed on the surface of the larval body, as commonly observed in competent larvae of regular echinoids. Some rudiments were oriented 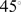, and not perpendicular, to the 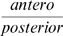 axis of the larva.

#### Starved

Compared to fed larvae, starved ones formed the same structures at similar developing times during the first week post-fertilization. Differences were noticed only during vestibule invagination. The vestibule of starved larvae began to invaginate, but did not reach the left coelom; larvae died 17 dpf.

### Metamorphosis

**Figure 10:**
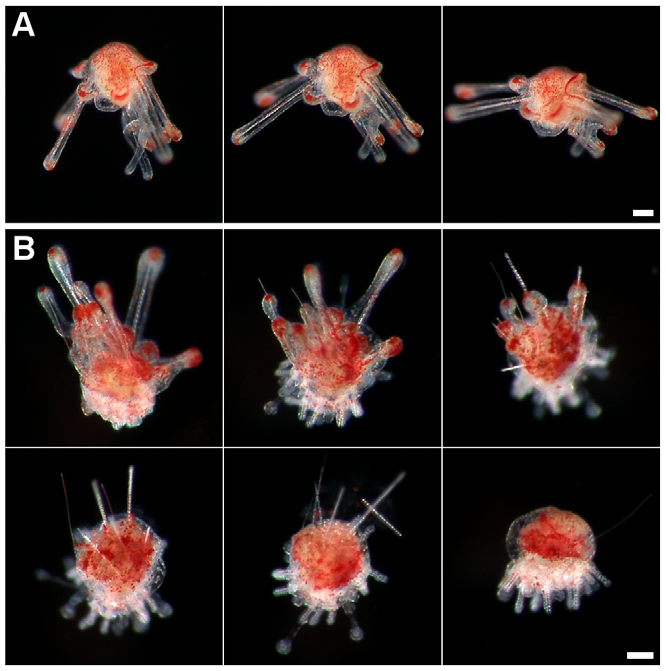
Substrate-test behaviour and metamorphosis of *C. subdepressus*. **A** Competent larvae opening the arms and exposing the vestibule pore. **B** Metamorphosis took approximately 1 h 30 min, from attachment to the complete regression of larval tissues. Scale bars = 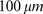

**Figure 11:**
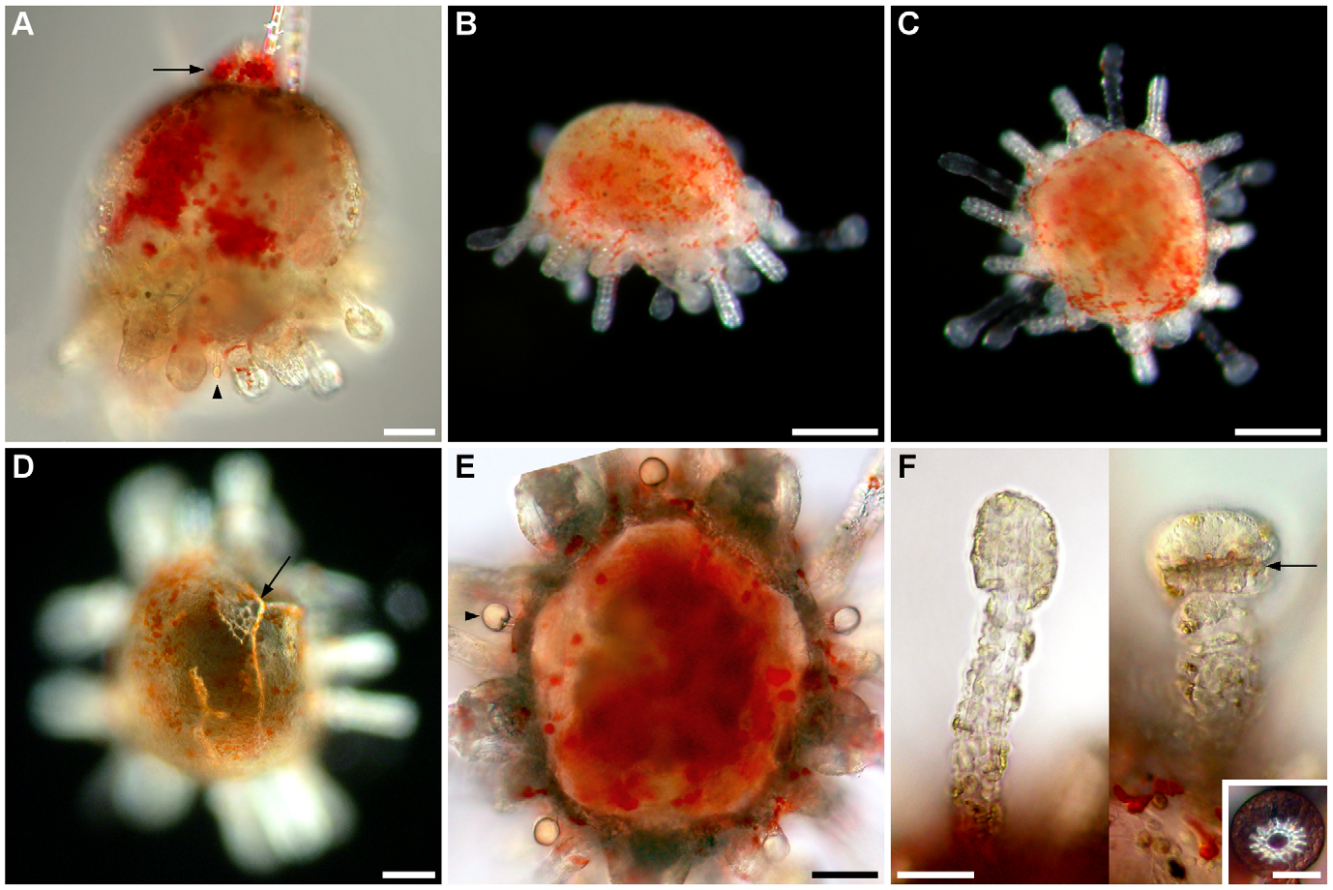
Morphology of postlarval juveniles of *C. subdepressus*. **A** Side view of the final stage of larval tissue regression; fragments of tissue were disrupted (arrow) near the skeleton of larval arms; a tiny sphaeridium (arrowhead) is visible between spines. **B** Side view showing the accumulation of larval tissues in the aboral region before the resorption. **C** Aboral view of **A**; bilaterality is not yet clearly identified. **D** Remnants of the larval skeleton (arrow) on the aboral surface under polarized light. **E** Distribution of sphaeridia (arrowhead) on the oral surface. **F** Different types of podia present after metamorphosis; type I (left) and type II (right) podia; circular spicule (arrow and detail). Scale bars = 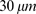 (**F**); 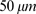 (**A, E**); 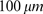 (**D**); 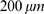 (**B, C**)

Competent larvae exhibited a typical substrate-test behaviour which consisted of swimming near the bottom and exposing the vestibule pore with protruding podia (type I, see below) by moving the left arms (Fig. 10A; Video S6). Larvae were able to fully evert the rudiment by opening the arms 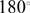 posteriorly, attach to the substrate, return to the larval conformation and resume swimming. Metamorphosis occurred when larvae attached firmly to the bottom with the protruding podia and the larval tissues began to regress and accumulate on the aboral surface of the rudiment (Fig. 10B). During this process larval spicules became exposed and broke off; fragments of tissue were lost (Fig. 11A). Larval tissues accumulated on the aboral surface of the rudiment forming a globoid structure (Fig. 11B). Metamorphosis took approximately 1 h 30 min from attachment to the complete regression of the larval tissues (Video S7).

### Juvenile

**Figure 12:**
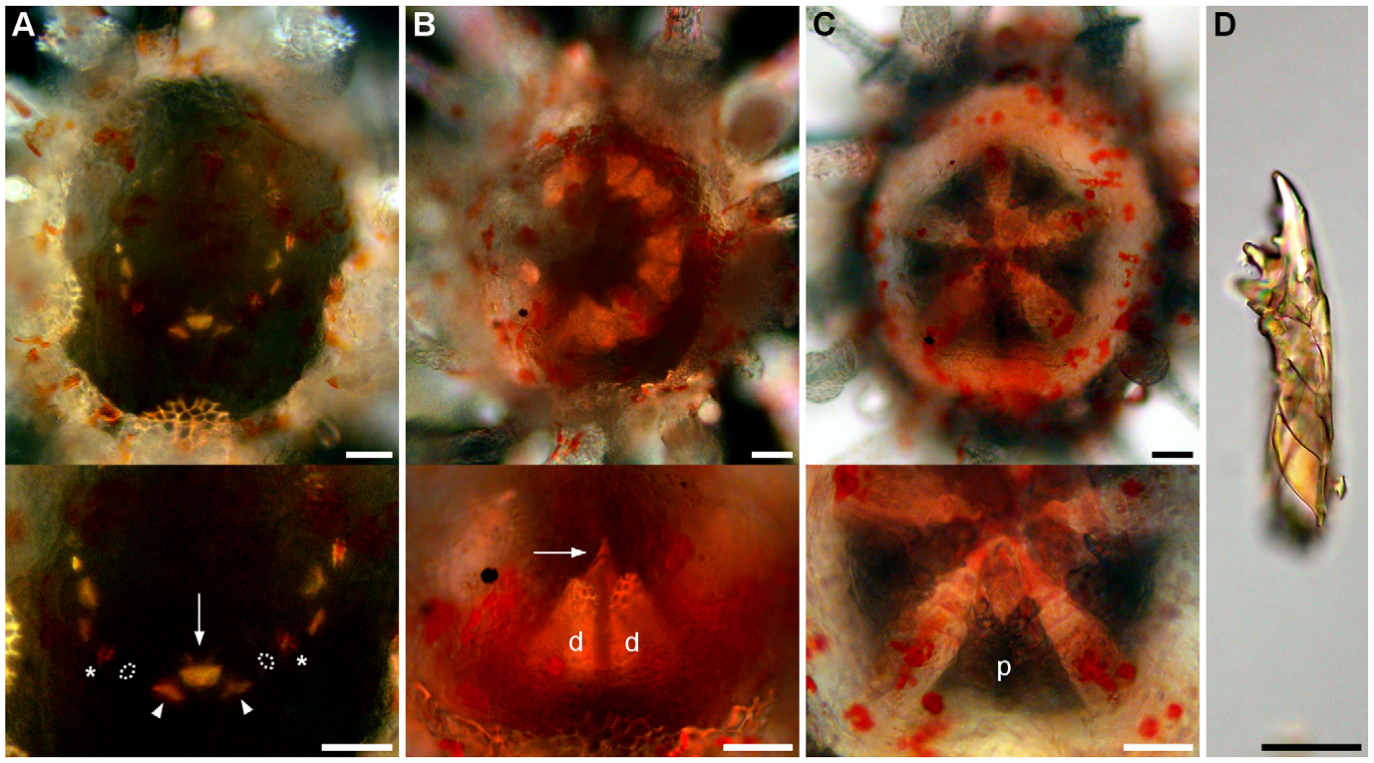
Formation of the lantern of Aristotle of *C. subdepressus*. **A** Rudiments of the lantern after the regression of larval tissues at the end of metamorphosis. Each set of ossicles was intercalated by a rotula (not visible) and consisted of a central tooth (arrow), two demipyramids (arrowheads), and two epiphyses (dashed line). Latter and rotula not visible because of orientation of polarized light. Asterisks mark the epiphyses of the adjacent sets. **B** Demipyramids (d) were formed, but not sutured together 2 dpm; teeth had narrow tips (arrow). **C** Demipyramids were tightly sutured into a pyramid (p) 7 dpm. **D** Mature teeth with ornamentation on the inner side (left). Scale bars = 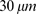

Metamorphosis is followed by the resorption of larval tissues and the development of juvenile structures. The body of metamorphosing and early postlarval juveniles did not exhibit clear evidence of bilateral symmetry when seen from the aboral surface (Fig. 11B). However, a close look at the oral surface under polarized light revealed hints of bilateral symmetry identifiable by the shape of the body and position of the rudiments of the lantern of Aristotle (Fig. 12A). The bilateral symmetry passing through the III-5 plane (Lovén's axis) became evident after the resorption of larval tissues. The bilaterality of postlarval juveniles was identified by the ovoid body shape, the longer interambulacrum-5 pair of posterior spines, the anus opening posteriorly, and the ambulacrum-III always pointing in the moving direction of the juvenile.

**Figure 13:**
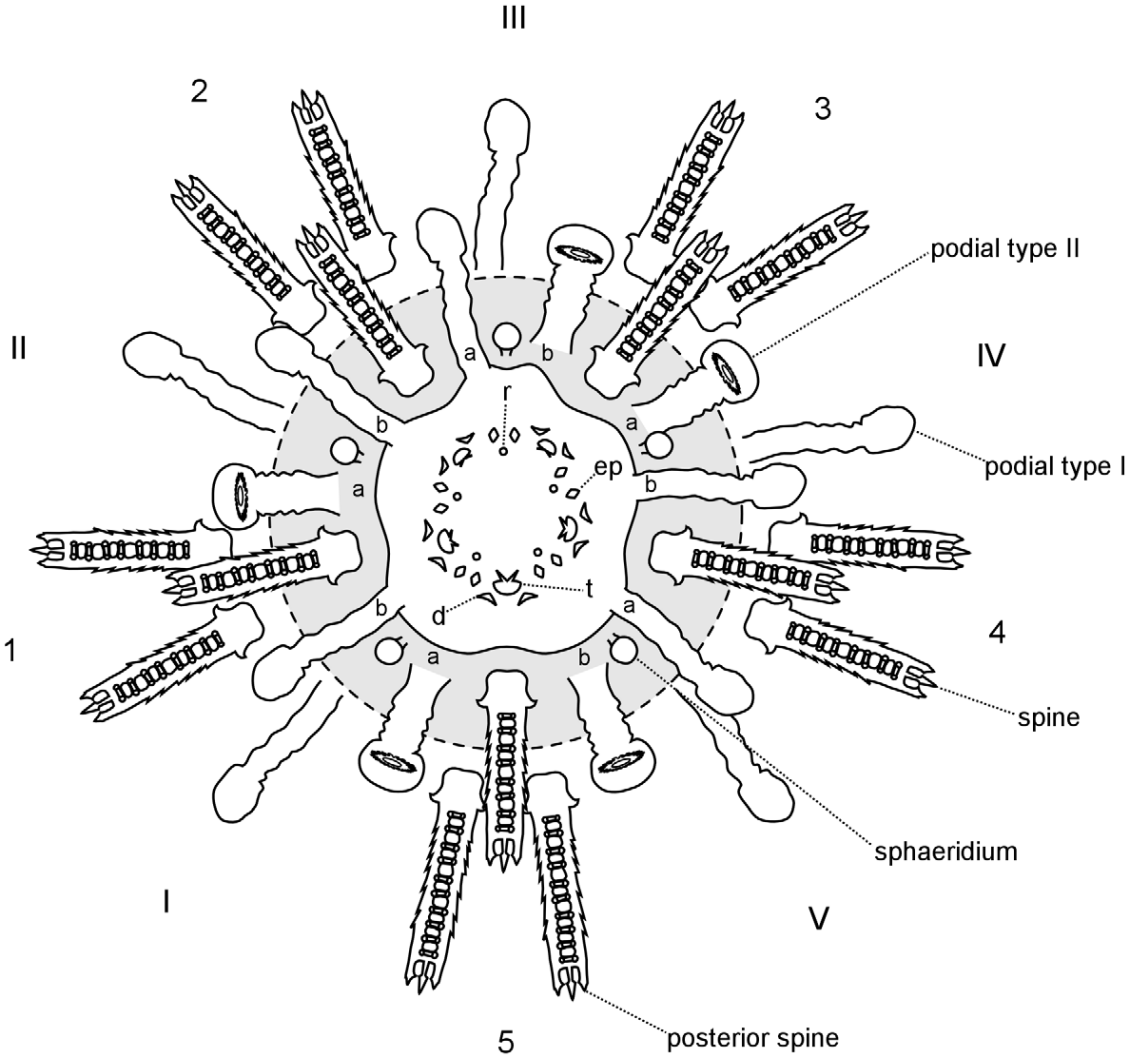
Postlarval appendages and lantern rudiments. Oral view representation of a postlarval juvenile after the regression of larval tissues. Ossicles of the lantern of Aristotle ossicles were present at the center of the oral region. Teeth (t), demipyramids (d), epiphyses (ep), and rotulae (r). Podia, spines, and sphaeridia are present on the calcified oral region (grey area). Dashed line delimits the second row of appendages positioned above the ambitus. Miliary spines not shown.

**Figure 14:**
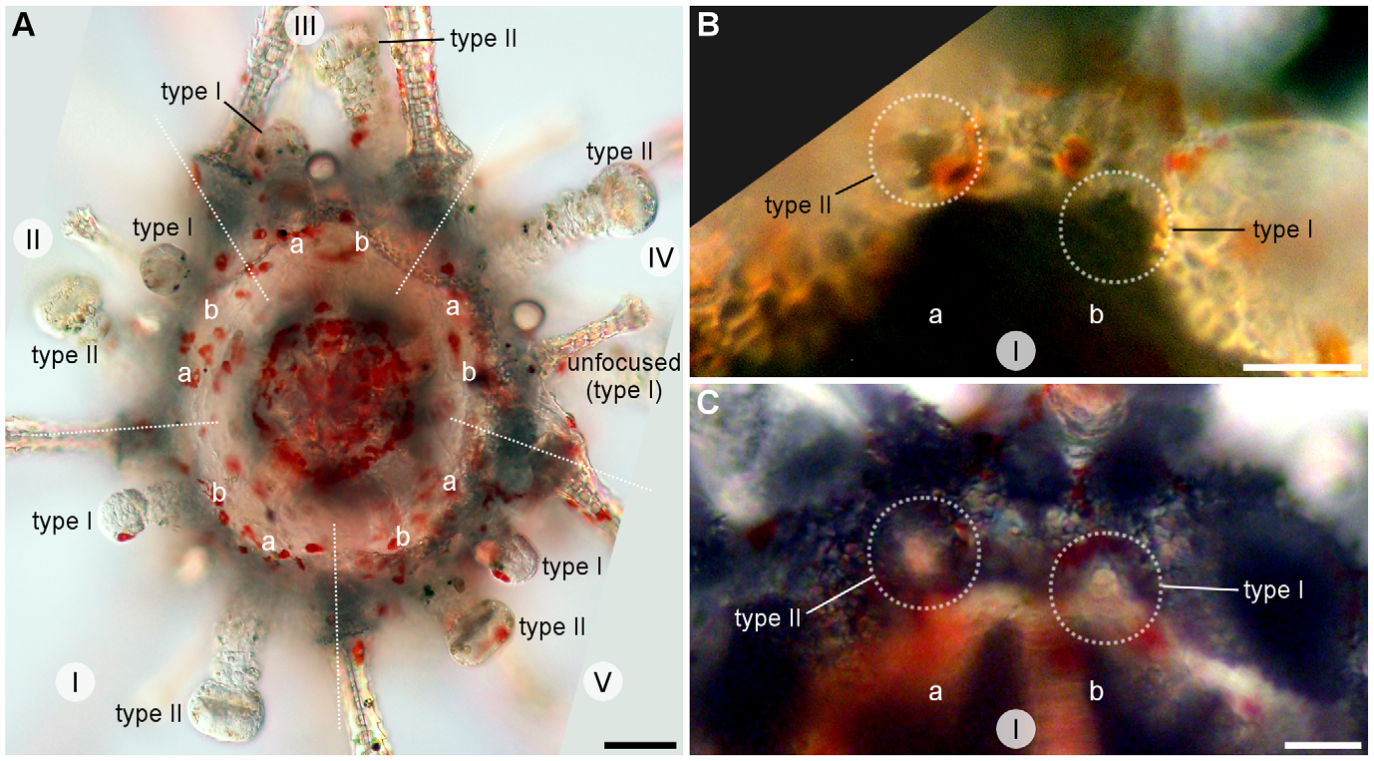
Ambulacra of *C. subdepressus* juveniles. **A** Oral view of a juvenile showing the distribution of podial types within ambulacral zones (I–V in white circles); *bbaba* for type I and *aabab* for type II. The middle section of interambulacra are marked with dashed white lines. **B** Detail of ambulacrum I of a juvenile during the resorption of larval tissues. Dashed white circles mark the position of the first two podia. Type I originated on the ambulacral column Ib on the peristome edge and was not encircled by the skeleton; type II emerged from a pore in the skeleton on the ambulacral column Ia. **C** Ambulacrum I of a 2 months old juvenile still showing the same pattern of the younger juvenile in **B**. Scale bars = 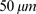 (**A**); 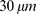 (**B, C**)

Appendages of 1-day-old postlarval juveniles were disposed in two rows along the ambitus, an infracoronal (oral) and a supracoronal (aboral) row. The infracoronal row had one spine for each interambulacral zone and one sphaeridium (a modified spine of uncertain function) between a pair of accessory podia for each ambulacral zone, totalling 5 sphaeridia, 5 spines, and 10 podia on the oral surface (Fig. 13). There were two types of suckered podia in postlarval juveniles; no buccal podia were observed. Type I had a narrow tip without an internal spicule (Fig. 11F, left) while type II had an expanded tip and a ring-shaped spicule inside (Fig. 11F, right). The former originated in the outer margin of the peristome (inner margin of the skeleton of the oral surface), but not embraced by the skeleton at ambulacral positions Ib, IIb, IIIa, IVb, and Va (*bbaba*; see Fig. 13 and 14A); podia was positioned in a concavity of the skeleton (Fig. 14B,C). The latter, podial type II, emerged from a skeleton pore at the complementary ambulacral columns Ia, IIa, IIIb, IVa, and Vb (*aabab*). Podial type I occupied an inner (closer to the mouth) ambulacral position than podial type II on the infracoronal row of juveniles (Fig. 14B,C). At this stage, sphaeridia were equal in size bearing a birefringent oval tip 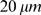 in diameter (Fig. 11E), but they were smaller during metamorphosis (width between 3 and 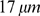; see Fig. 11A). The supracoronal row had a pair of spines for each interambulacral area and a type I podium for each ambulacral zone. The postlarval juvenile had 15 spines, 15 podia, and 5 sphaeridia in total; miliary spines were occasionally present on ambulacral zones and no pedicellariae were observed at this stage. Primary spines and podia of *C. subdepressus* were permanent and did not regress during the observed period.

Early postlarval juveniles had no skeleton on the aboral surface, except for the remnants of larval rods (Fig. 11D). The gut was not yet formed and neither mouth nor anus were present. During the resorption of larval tissues the rudiments of the lantern of Aristotle were visible in the oral region under polarized light. Teeth were positioned on the interambulacral zones between a pair of demipyramids and a pair of epiphyses (Fig. 12A). Each set of 5 independent ossicles was intercalated by a single ossicle, a rudiment rotula (Fig. 13). Except for the teeth, the skeleton had the trabecular structure typical of stereom.

**Figure 15:**
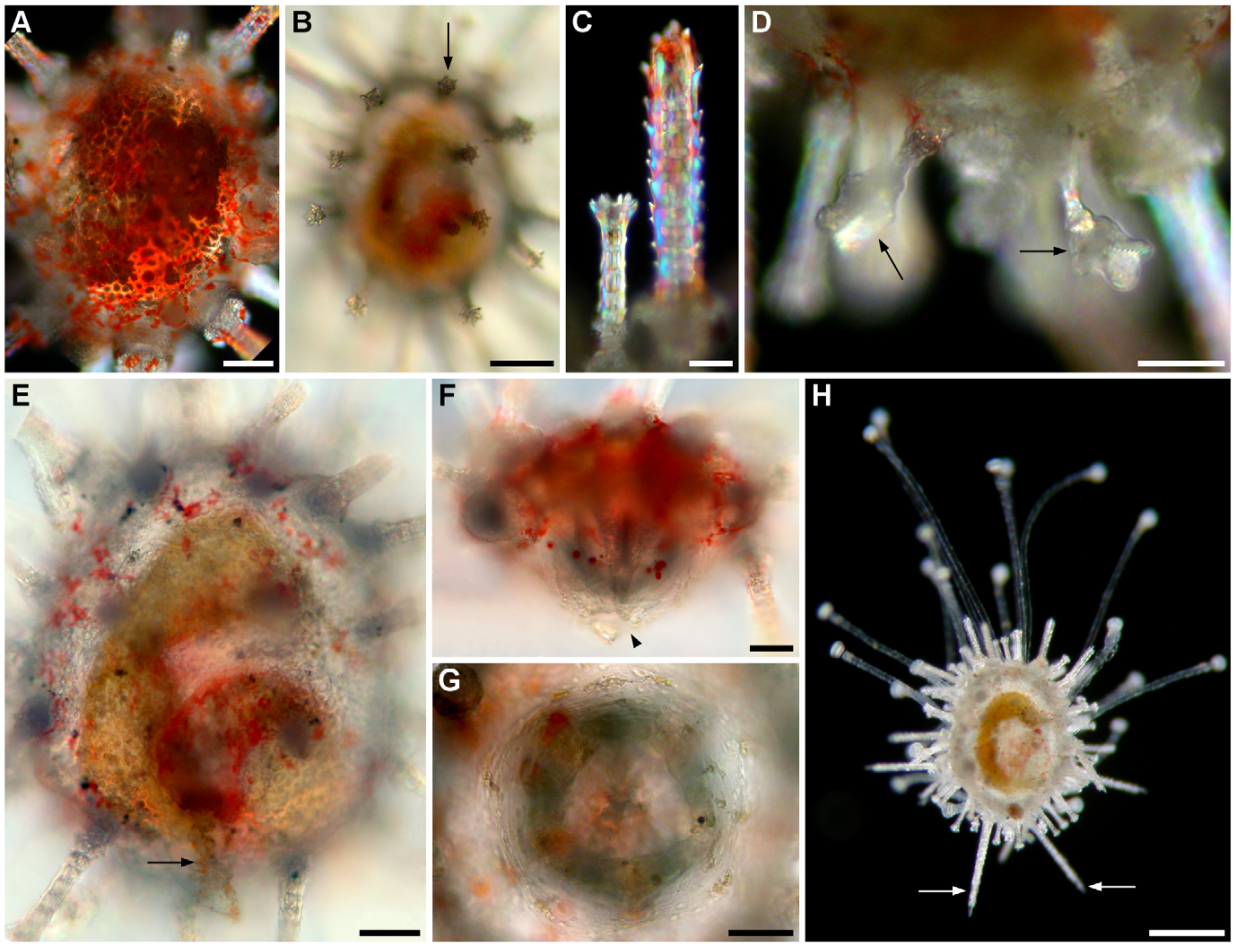
Aboral structures and mouth formation of *C. subdepressus*. **A** Skeletal plates on the aboral surface of a 2 d old juvenile under polarized light. **B** Aboral view showing the distribution of miliary spines (arrow). **C** Detail of a miliary (left) and primary (right) spine. **D** Pair of ophicephalous pedicellariae (arrows) at the posterior region of the juvenile. **E** Digestory track of the juvenile and fecal pellet (arrow) being released by the anus. **F** Side view of the mouth (arrowhead) after the opening of the peristomial membrane. **G** Oral view showing the mouth and peristomial membrane 7 dpm. **H** Juvenile 120 dpf during locomotion with podia extended anteriorly and the characteristic longer pair of posterior spines (arrows). Scale bars = 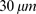 (**C, G**); 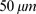 (**A, D, E, F**); 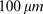 (**B**); 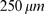 (**H**)

Two days after metamorphosis the demipyramids were larger and still separated by the tooth slide (Fig. 12B). Tips of the teeth were narrow without ornamentation (Fig. 12B) and became robust with a complex surface 4 dpm. The digestive tract became visible through the aboral surface two days after metamorphosis; the gut tube exits the lantern of Aristotle onto the right side of the cavity and towards the anterior region; it turned counterclockwise towards the left side of the cavity, into the posterior region, and then again reaching the right side. The tube then bent 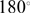 left, pointing posteriorly, and opened on the posterior portion of the aboral surface below an anal plate (Fig. 15E).

The aboral surface was occupied by calcareous plates 2 dpm (Fig. 15A). Eight miliary spines (with a crown-like tip) grew on the supracoronal row and 4 appeared on the aboral surface between 2 and 4 dpm (Fig. 15B,C); older juveniles also had miliary spines between other appendages, including ambulacral zones. Each pair of demipyramids were sutured together into one pyramid 7 dpm (Fig. 12C). No other skeletal element was formed on the oral surface of the juvenile during this period.

The mouth opened on the centre of the oral surface 7 dpm and juveniles began to feed (Fig. 15F,G). At this stage the peristomial membrane and the lantern of Aristotle were actively moving while the esophagus exhibited peristaltic contractions (Video S8). Exotrophic juveniles frequently had food in their gut and eventually in the rectum; fecal pellets were observed (Fig. 15E). Type II podia significantly increased in number and seemed to play a major role in locomotion. Juveniles moved quickly among sand grains, extending the anterior type II podia forward more than a body length ahead (Fig. 15H; see Video S9 for locomotory examples).

One-month-old juveniles exhibited a digestive tract with three distinct parts besides the esophagus. The stomach and intestine were wider, opaque, and brownish while the rectum was covered by red-pigmented cells (Fig. 15E). Four ophicephalous pedicellariae appeared on the ambitus of the posterior region 14 dpm (Fig. 15D). Tridentate pedicellariae appeared at the posterior region 30 dpm; they were approximately one quarter of the size of the posterior spines with a stem two thirds of the length. This kind of pedicellaria was also seen on the middle region of the juvenile, but not on the anterior portion.

Juveniles did not eat free living benthic organisms, but apparently rasped organic material off larger sand grains. Smaller particles were manipulated by podia and held in position by the peristomial membrane while the lantern rubbed the surface of the grain (Video S10). They survived in laboratory cultures for 8 months and 20 days after fertilization. Post-metamorphic juveniles were initially 200–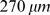 wide and 300–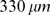 in length and reached 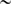 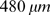 in diameter and 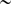 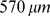 in length. Juveniles were active and showed no sign of food privation, except for the last 3 weeks when they became less active and more pale. A single sphaeridium per ambulacrum was still visible (not internalized) by the time of juvenile death.

## Discussion

Direct observations of the behavior of *C. subdepressus* during spawning revealed that specimens covered by a layer of sand (not completely buried) repeatedly exposed the apex, released gametes, and covered the apex by moving the grains adapically. Apex exposure before spawning was observed previously in burrowed individuals of *Arachnoides placenta* [17], but intermittent gamete release during spontaneous spawning was previously undescribed for clypeasteroids. Until there are field observations to better comprehend the external fertilization dynamics of clypeasteroids, their reproductive behavior will remain largely unknown.

Although it was suggested that long papillae, swollen during spawning, could disperse gametes across a thin layer of sand [17], our observations showed that *C. subdepressus* avoid contact of gametes with sand grains by clearing the apex during spawning. Thus, the genital papillae seem to play a role in allowing gametes to be released well above the spines, consequently decreasing the number of eggs and sperm trapped within the skin mucus [17] and avoiding mechanical stress caused by the movement of spines and pedicellariae.

Cleavages of *C. subdepressus* were similar to other echinoids with planktotrophic larvae [10], [18]–[20], except that macromeres were approximately 30% smaller than mesomeres. The timing of the formation and hatching of *C. rosaceus* blastulae took longer (12 h at 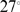
C [10]) than in *C. subdepressus* (7 h at 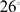
C). Developmental timing of later stages did not differ between Brazilian and Caribbean specimens of *C. subdepressus* at 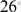
C and 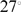
C, respectively [10].

Data on the gastrulation of *C. subdepressus* fits the correlation between the type of gastrulation and the pattern of migration of red-pigmented cells in echinoids [21]; red-pigmented cells originate on the vegetal pole and migrate through the ectoderm to the apical plate while the archenteron elongation is continuous. Also, the behaviour of red-pigmented cells matches the pattern described for *C. japonicus* where these cells migrate through the ectoderm, but are also seen at the archenteron tip [21]. Red-pigmented cells can have a regulatory role and are known to trigger gastrulation in *Echinometra mathaei* [22]. These cells might participate on the morphological changes during prism formation and early axis specification of plutei, because they were absent from the ventral ectoderm of *C. subdepressus*; a region that remained flat until the formation of the larval mouth.

Starved larvae of *C. subdepressus* neither develop a complete vestibule invagination nor a rudiment, but the eggs provided sufficient energy for the survival of a functional pluteus larva for 17 dpf. Based on larval skeleton measurements, the facultative feeding period of larvae was estimated in 5 clypeasteroid species [13]. The authors determined when fed and starved larvae differed in size from fertilization, indicative of the exhaustion of maternal provisioning. In *C. subdepressus* larvae diverged between 60 and 72 hpf. This range matches with the necessary timespan for the differentiation of the larval gut, ciliated bands and beginning of larval feeding, described in the present study. Thus, we directly observed that the pre-feeding stage of *C. subdepressus* larvae ends 3 dpf. The appearance of size differences between larvae might not solely indicate when starved larvae stopped growing, but also when fed larvae increased their growth rate because of food ingestion. Since starved larvae reached the 8-arm pluteus stage without qualitative morphological differences, the exhaustion of maternal resources in *C. subdepressus* apparently does not occur as early as suggested; even though the absence of food immediately after the pre-feeding stage could directly affect early growth rates.

Competent larvae of *C. subdepressus* exhibited a substrate-test behaviour similar to other echinoid species [23]–[26]. Although early postlarval juveniles resemble regular urchins with a spherical body, bilateral symmetry could be identified soon after the resorption of larval tissues and was probably determined during rudiment formation. Several morphological features mark the early bilateral symmetry of *C. subdepressus* such as elongated body, longer posterior spines, and anus positioned posteriorly. In other irregular urchins bilateral symmetry is recognized by different characters. In the spatangoid *Echinocardium cordatum*, the subanal fasciole is visible on the rudiment still inside the larvae [26], the peristome is positioned anteriorly and the posterior region is more developed [27]. The bilateral symmetry of the sand dollar *Echinarachnius parma* is identified by the size and number of plates in the ambulacra and by differences in size of the sphaeridia [28].

The *bbaba* distribution at ambulacral columns and the inner position of infracoronal type I podia suggests strict adherence to Lovén's rule of echinoid peristome development [29]. These patterns indicate that *C. subdepressus* podial type I preceded type II during ontogeny. Since we did not track the formation of skeletal plates, the coordinance between the appearance of podia and growth of skeletal plates has not been elucidated. We observed that the first podium (type I) was not encircled by the skeleton and occupied the edge of the peristome, while the following podium on the ambulacrum (type II) protruded from a pore in the calcareous mesh. At this early stage there were no signs of disruption in the alternating pattern of podial organization, which in other echinoids is a by-product of the alternating deposition of plates known as the “ocular plate rule” [30]. Therefore additional data on juvenile skeletal growth remains crucial to trace the plate formation and ontogenetic fate of these podia. In addition, precise tracking of ambulacral growth can identify the developmental basis behind a well-known innovation of Clypeasteroida, the breakage of the one-podium-per-plate rule and further increase in the number of nonrespiratory podia [2].

Following metamorphosis *C. subdepressus* juveniles had 3 spines per interambulacrum (15 total), 3 podia and one spheridium per ambulacrum (15 podia and 5 sphaeridia total), and no pedicellariae. In contrast, competent larvae of the regular urchins *Paracentrotus lividus* and *Strongylocentrotus franciscanus* already have pedicellariae during the late larval period and after metamorphosis [25], [31], while pedicellariae of *S. purpuratus* appear some time after metamorphosis. Competent larvae of *E. cordatum* do not exhibit spines or pedicellariae [26]. Differently from *C. subdepressus*, newly metamorphosed juveniles of these regular sea urchins have 4 primary spines per interambulacrum (20 total) [25], [31]. The irregular echinoid *E. cordatum* has a greater number of primary spines per interambulacrum after metamorphosis and also differs from *C. subdepressus* by the presence of secondary spines and a subanal fasciole with clavulae and 4 primary spines [26]. By contrast, *C. subdepressus* uniquely displays 3 podia per ambulacrum after metamorphosis while *S. franciscanus* and *S. purpuratus* [31], *P. lividus* [25] and *E. cordatum* [26] have 1 podium per ambulacrum. The two podial types identified in postlarval juveniles of *C. subdepressus* did not clearly fit into any specific category previously described for adult clypeasteroids [32]. Further podial specialization should occur associated with body growth and differentiation of food grooves. The appendages of *C. subdepressus* did not regress after metamorphosis as observed for *E. cordatum*, but the postlarval period of *C. subdepressus* (7 d to exotrophy) was considerably longer than observed for *E. cordatum* (3.5 d) [26]; regular echinoids have a longer postlarval period (8–10 d) [25], [31]. Differences in nutrient storage and latent effects of larval life [33] might account for variations in the postlarval period. Finally, the mouth of *E. cordatum* opens before the anus while the mouth of *C. subdepressus* opened after the anus.

The initial formation of the lantern of Aristotle of *C. rosaceus* was identified between 24 and 48 h; although it became functional 7 d later, its development was completed in approximately 12 d, when the mouth opened [10]. In contrast, the rudiments of the lantern of *C. subdepressus* were already present during metamorphosis, and feeding began soon after (7 dpf). At this early stage, size obviously poses limitations to the feeding mechanisms of juveniles. While adult *C. subdepressus* systematically collects and grinds sand grains [9], juveniles actively harvested individual grains using the lantern of Aristotle as a rasping tool. The strength to crush sand grains and the ability to collect many particles simultaneously depend on a larger lantern of Aristotle and a greater number of podia available, respectively; both are expected to be acquired with body growth. In other words, size can directly shape the feeding habits of developing sea biscuits.

Adult specimens of Cassiduloida and Oligopygoida, ancestral lineages of the Clypeasteroida, have multiple open sphaeridia per ambulacrum [2]. The Clypeasterina has 2 closed sphaeridia per ambulacrum while the remaining clypeasteroids (Laganiformes and Scutelliformes) have only 1 closed sphaeridium. Although a complete ontogeny of sphaeridia is known only for isolated species, it is known that all irregular echinoids have a single open sphaeridium per ambulacrum after metamorphosis [2]. The spatangoid *E. cordatum* bears 5 sphaeridia on the ambulacral zones after metamorphosis [26], [34] like *E. parma* [28] and *C. subdepressus* (present work). A subtle difference between spatangoids and clypeasteroids is that sphaeridia of the latter are not equal in size during the resorption of larval tissues after metamorphosis. This might indicate that the rudiments of spatangoids are more developed at metamorphosis. Regular urchins exhibit calcified sphaeridia only 9 d after metamorphosis in both *S. franciscanus* and *S. purpuratus* [31] and 6 d after metamorphosis in *P. lividus* [25], even though a sphaeridial bud grows after 4 d in the former. The sphaeridia remained open for nearly 9 months showing that enclosure does not occur shortly after metamorphosis in *C. subdepressus* as previously suggested for Clypeasteroida [2]. We have no further evidence to suggest that enclosure of sphaeridia occurs before or after the appearance of the second sphaeridium, which is present in adult specimens of Clypeasterina.

We found only 3 small-sized specimens and no individuals less than 8 cm in diameter at sampling sites (personal observation). The occasional nature of recruitment events of *C. subdepressus* might affect their size distribution in the field. Also, the cryptic habit, high mortality [35] and the minute size of newly metamorphosed juveniles greatly decreases the probability of finding them in their natural habitat.

Exotrophic juveniles of *C. subdepressus* raised in laboratory cultures for nearly 9 months did not grow more than 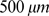 across. Accordingly, post-metamorphic juveniles of *C. subdepressus* and *C. rosaceus* from the Caribbean increased in diameter for 8 d and then ceased growth at approximately 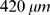 [10]. Although *C. subdepressus* juveniles from São Sebastião survived considerably longer than the 30 d observed for the Caribbean specimens, the conditions of death were similar; juveniles progressively became more transparent with slower movements. Since juveniles frequently had food in the gut and released fecal pellets, they could have suffered a progressive nutritional deficiency because of the limited conditions of laboratory cultures. The absence of essential nutrients and/or appropriate diet might have inhibited their further development. To explain the minimal growth, one could suggest that *Clypeaster* species have inherently slow growth rates and a long life span. However, this hypothesis still needs to be properly tested once we learn more about the feeding habits and ideal habitat conditions for the growth and survival of juveniles. Until then, a series of morphological changes occurring between the juvenile and adult life, such as the anus migration to the oral surface, the appearance of the second sphaeridium, the enclosure timing of sphaeridia within the test, and associated shape changes, will remain undocumented in these large clypeasteroids.

## Materials and Methods

### Collecting adults

**Figure 16:**
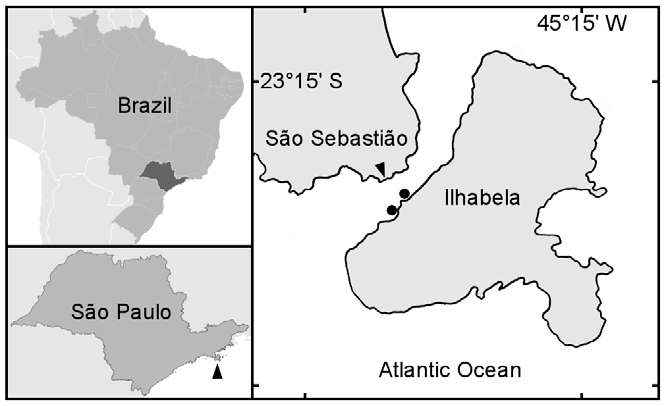
Collecting sites of adult *C. subdepressus*. Portinho and Parcel da Praia Grande (dots) are located on the island shore of São Sebastião Channel. CEBIMar-USP (arrowhead) is located on the continent shore at the Northern region of São Paulo State. Marks every 15′. Brazil and São Paulo vector images cortesy of Felipe Menegaz and Raphael Lorenzeto de Abreu, respectively.

We collected adult specimens of *Clypeaster subdepressus* Gray, 1825 on sand beds 4–6 m deep in São Sebastião Channel, Northern shore of State of São Paulo, Brazil. We sampled between January and March 2007 at two locations: Portinho (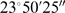 S; 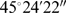 W) and Parcel da Praia Grande (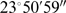 S; 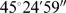 W) (Fig. 16). We kept the specimens in 500 L tanks with continuous flowing sea-water and sediment from collecting sites at Centro de Biologia Marinha da Universidade de São Paulo (CEBIMar-USP).

### Cultures

#### Embryos and larvae

We induced spawning by puncturing the medial portion of one food groove (ambulacrum III) and injecting 2–3 mL of 0,53 M KCl solution into the body cavity. Normally, a second injection of 2 mL was required to trigger gamete release. We poured the eggs through a 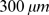 nylon mesh and suspended them in a 500 mL beaker with filtered sea-water (FSW). Eggs were fertilized with 1 mL solution of diluted sperm and moved to a 4.5 L bowl with FSW. After fertilization we kept cultures at 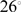
C and natural light. Approximately 10 h post fertilization (hpf) we transferred swimming larvae to vessels of 100 mL with FSW and no aeration. We kept a maximum of 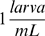 and inspected the cultures daily. During these observations we removed dead larvae and changed 80% of the sea water with a plastic pipette and a 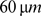 nylon mesh, or transferred healthy larvae to a new vessel.

When larvae began to feed we added *Dunaliella tertiolecta* and *Rhodomonas* sp. to the cultures daily at a final concentration between 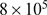 and 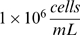. Ten cultures were deprived of food, but received a normal maintenance routine (i.e. water changes).

#### Metamorphosis

We transferred advanced pluteus larvae to petri dishes with FSW and added a few sand grains from the sampled sites. Successfully metamorphosed larvae were kept in the same container overnight and then moved to a different culture, shown below.

#### Juveniles

We kept juveniles in the same 100 mL vessels used for larvae, but with a monolayer of sand on the bottom. We changed the water weekly and sealed the cultures with PVC film to minimize water evaporation and subsequent rise in salinity. During water changes we added 3 mL of a plankton sample or biofilm of aquaria, in an attempt to keep food (organic content) available for the juveniles, once we did not change the substrate of these cultures.

### Kinetics of cleavages

We homogenized the suspension of developing embryos and collected 2 samples of 3 mL in 10% sea-water formalin every 20 min during a period of 180 min. Posteriorly, we counted the total number of embryos (sum of samples) and estimated the relative frequency of each stage, according to the number of cells (1, 2, 4, 8, 16 and 32 cells), for each timespan.

### Archenteron elongation during gastrulation

To verify if the gastrulation of *C. subdepressus* was continuous or truncated we fixed samples of developing embryos every 30 min between 12 and 20 hpf (except 12.5 hpf) in 1% paraformaldehyde. For each period we transferred the embryos to glass slides, photographed, and measured their width, height, blastocoel height and archenteron length (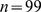) with the image processing software ImageJ [36]. The final degree of invagination was calculated with the ratio between the archenteron length and blastocoel height.

### Light microscopy

We documented the development under differential interference contrast (DIC) with a Nikon Coolpix 4500 for photomicrographs and Sony DCR HC1000 digital video camera for videos. Cameras were attached to a Zeiss Axioplan2 compound scope and a Zeiss Stemi SV11 APO stereomicroscope. We visualized the calcareous elements of larvae and juveniles under polarized light.

#### Pluteus larva reconstruction

After manually capturing 118 sequential focal planes of a pluteus larva under DIC we imported the images into ImageJ [36]. The stack was converted to 8-bit, aligned, inverted, and the voxel depth set to 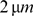. We corrected the contrast/brightness so that only areas in focus of each image remained visible. Finally, we created a maximum intensity z-projection (Fig. S1A) and a red-cyan anaglyph of the larva (Fig. S1B).

### Scanning electron microscopy

We fixed samples in 2% glutaraldehyde, post-fixed in 1% osmium tetroxide, and dehydrated in ethanol series until 100% ethanol. Standard procedures of critical point and metal coating were done at Instituto de Biociências da Universidade de São Paulo.

## Supporting Information

Figure S1 Frontal view of a pluteus larva of *Clypeaster subdepressus* reconstructed from differential interference contrast image-sequence. A Grayscale reconstruction showing the position of the arms. B Red-Cyan 3D image of the same pluteus larva. Scale bar = 50 µm
(5.02 MB TIF)

Video S1 Female of the sea biscuit *Clypeaster subdepressus* releasing eggs through the gonopore papilla.
(1.43 MB AVI)

Video S2 Main and accessory papillae of a male sea biscuit *Clypeaster subdepressus* releasing sperm.
(1.38 MB AVI)

Video S3 Elevation of the fertilization membrane after sperm entry, pronuclei migration, and initial clevages of *Clypeaster subdepressus*.
(5.36 MB AVI)

Video S4 Pre-feeding early pluteus larva rejecting microalgae.
(0.35 MB AVI)

Video S5 Esophagus initial contractions of a pre-feeding early pluteus larva.
(0.89 MB AVI)

Video S6 Pluteus larvae with a well-developed rudiment exhibiting the “substrate test behavior”. Larvae open the arms exposing the rudiment while podia touches the substrate. If metamorphosis is not initiated larvae can resume swimming.
(5.98 MB AVI)

Video S7 Metamorphosis of the sea biscuit *Clypeaster subdepressus*.
(2.00 MB AVI)

Video S8 Movements of the lantern of Aristotle, the peristome, and the esophagus of a *Clypeaster subdepressus* feeding juvenile.
(2.60 MB AVI)

Video S9 Activity and locomotion of postlarval and feeding juveniles.
(5.24 MB AVI)

Video S10 Feeding juvenile manipulating a tiny sand grain with podia and peristome.
(2.99 MB AVI)
