# Supplementary figures and images for: Embryonic, Larval, and Juvenile Development of the Sea Biscuit Clypeaster subdepressus (Echinodermata: Clypeasteroida)
Source: PLoS One. 2010 Mar 22;5(3):e9654. doi: 10.1371/journal.pone.0009654 (PMC2842294; doi:10.1371/journal.pone.0009654)

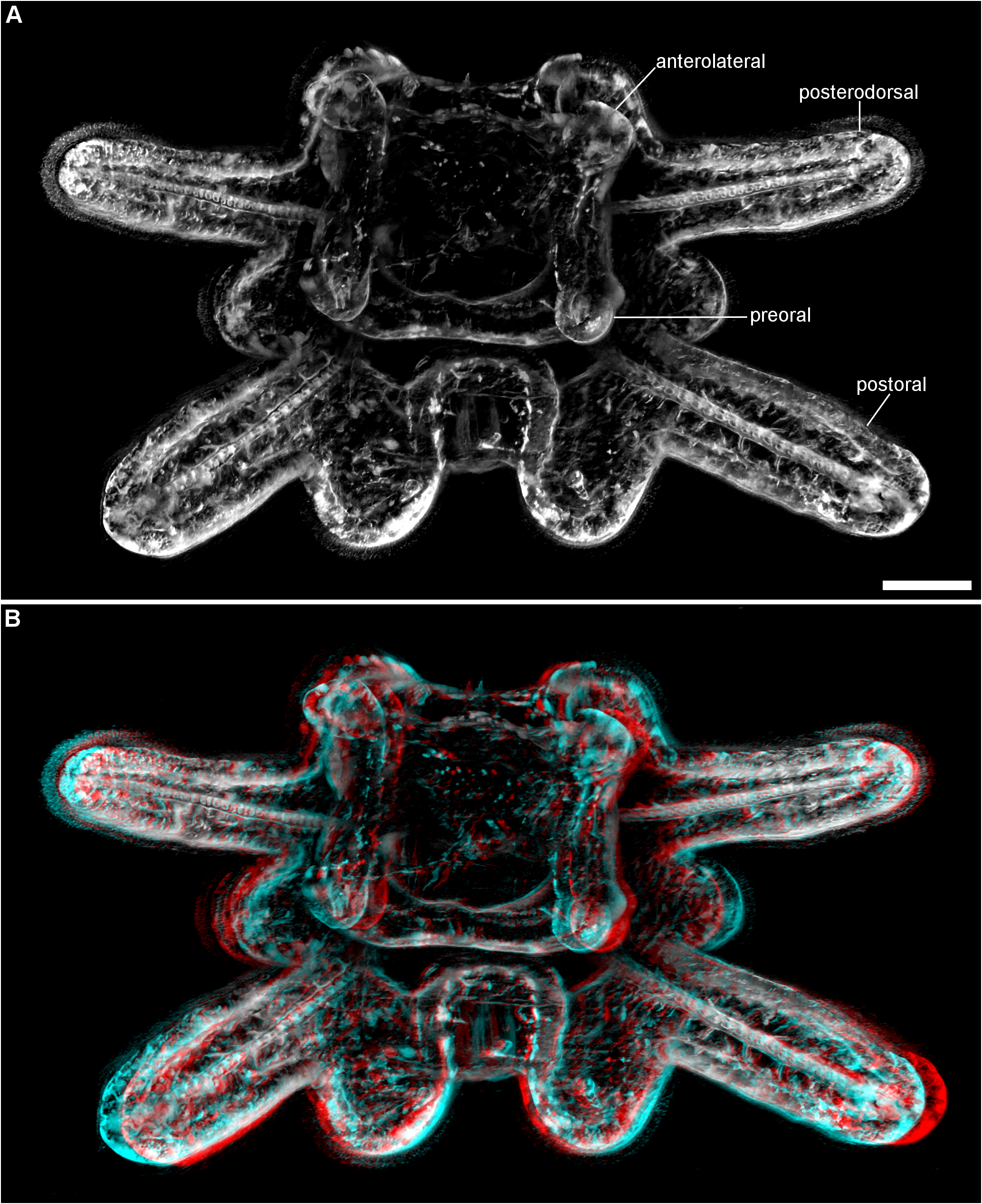

Supplement: Figure S1 — Frontal view of a pluteus larva of Clypeaster subdepressus reconstructed from differential interference contrast image-sequence. A Grayscale reconstruction showing the position of the arms. B Red-Cyan 3D image of the same pluteus larva. Scale bar = 50 µm (5.02 MB TIF) [file pone.0009654.s001.tif]
